# Correction: Isa et al. HSV-1 ICP22 Is a Selective Viral Repressor of Cellular RNA Polymerase II-Mediated Transcription Elongation. *Vaccines* 2021, *9*, 1054

**DOI:** 10.3390/vaccines12040354

**Published:** 2024-03-26

**Authors:** Nur Firdaus Isa, Olivier Bensaude, Nadiah C. Aziz, Shona Murphy

**Affiliations:** 1 Sir William Dunn School of Pathology, University of Oxford, Oxford OX1 3RE, UK; 2 Research Unit for Bioinformatics and Computational Biology, Department of Biotechnology, Kulliyyah of Science, International Islamic University Malaysia, Kuantan 25200, Pahang, Malaysia; nadiah.aziz@live.iium.edu.my; 3 Ecole Normale Supérieure, Institut de Biologie de l’Ecole Normale Supérieure, PSL Research University, CNRS UMR 8197, INSERM U 1024, F-75005 Paris, France; bensaude@bio.ens.psl.eu

The authors would like to make the following corrections to this published paper [1].

Upon reviewing Figure 3C (lane Y230), the authors observed a formatting error in the blot. Thus, Figure 3 should be replaced with the following version:

**Figure 3:**
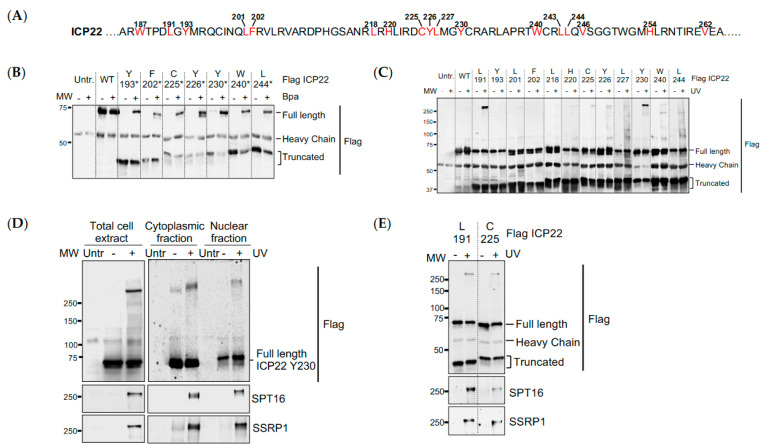
**UV crosslinking reveals that ICP22 interacts directly with the FACT complex.** (**A**) Amino-acid sequence of ICP22 showing the positions at which a TAG stop codon is introduced and Bpa is incorporated within ICP22; (**B**) HEK293 cells were co-transfected with wild-type ICP22 cDNA (WT) or ICP22 cDNA with a TAG stop codon replacing different amino acid codons (represented by the asterisk) and suppressor tRNA/Bpa synthetase pair in the absence or presence of photo-cross-linkable Bpa. Flag immunoprecipitation was performed from total cell extracts and separated by SDS-PAGE. Western blots were probed with an anti-Flag antibody; (**C**) Flag immunoprecipitation of ICP22^Bpa^ from cell extracts after cells were UV irradiated alive (+) in chilled PBS or not (−). Western blot was probed with an anti-Flag antibody; (**D**) HEK293 cells were co-transfected with ICP22 cDNA with a TAG stop codon replacing Y230 and suppressor tRNA/Bpa synthetase pair in the presence of photo-cross-linkable Bpa, and UV-irradiated alive (+) in ice-cold PBS. Flag immunoprecipitation was performed from total cell extracts, cytoplasmic and nuclear fractions and separated by SDS-PAGE. Western blots were probed with antibodies against Flag and FACT complex subunits SPT16 and SSRP1; (**E**) HEK293 cells were co-transfected with ICP22 cDNA with a TAG stop codon replacing L191 or C225 and suppressor tRNA/Bpa synthetase pair in the presence of photo-cross-linkable Bpa, and UV-irradiated alive (+) in ice-cold PBS. Flag immunoprecipitation was performed from total cell extracts and separated by SDS-PAGE. Western blots were probed with antibodies against Flag and FACT complex subunits SPT16 and SSRP1.

The authors apologize for any inconvenience this may have caused and affirm that the scientific conclusions remain unaffected. The original publication has also been updated.

## References

[1] Isa N.F., Bensaude O., Aziz N.C., Murphy S. (2021). HSV-1 ICP22 is a selective viral repressor of cellular RNA polymerase II-mediated transcription elongation. Vaccines.

