# Supplementary material for: HSV-1 ICP22 Is a Selective Viral Repressor of Cellular RNA Polymerase II-Mediated Transcription Elongation
Source: Vaccines (Basel). 2021 Sep 22;9(10):1054. doi: 10.3390/vaccines9101054 (PMC8539892; doi:10.3390/vaccines9101054)
Supplement: Supplementary file 1 [file vaccines-09-01054-s001.zip › Supplementary Figure S1.pdf]

Supplementary Figure S1

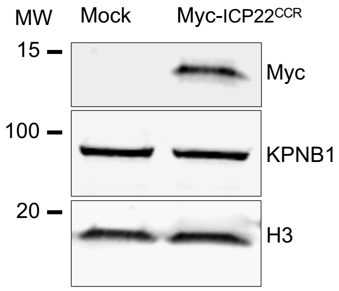

Western blot analysis of KPNB1 (Primary antibody A300-482A; Bethyl lab) in HEK293 cell extract after transfection of a vector expressing Myc-tag ICP22^{CCR}. H3 is used as loading control.
