# Supplementary material for: HSV-1 ICP22 Is a Selective Viral Repressor of Cellular RNA Polymerase II-Mediated Transcription Elongation
Source: Vaccines (Basel). 2021 Sep 22;9(10):1054. doi: 10.3390/vaccines9101054 (PMC8539892; doi:10.3390/vaccines9101054)
Supplement: Supplementary file 1 [file vaccines-09-01054-s001.zip › Supplementary Figure S2.pdf]

Supplementary Figure S2

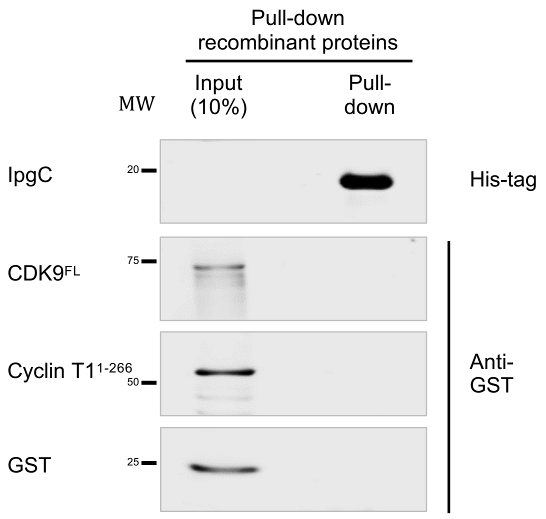

His-IpgC were bacterially expressed and immobilized on nickel magnetic beads. GST-Cyclin T1$^{1\text{-}266}$ and GST-CDK9^{FL} were expressed separately in *E.coli*. Immobilized GST-Cyclin T1$^{1\text{-}266}$ and GST-CDK9^{FL} were eluted with reduced glutathione, and incubated with on-bead His-IpgC. Both input and proteins retained on the beads after washes were subjected to western blot with GST and His-tag antibodies. Input represents 10% of that used for pull-down.
