# Supplementary material for: HSV-1 ICP22 Is a Selective Viral Repressor of Cellular RNA Polymerase II-Mediated Transcription Elongation
Source: Vaccines (Basel). 2021 Sep 22;9(10):1054. doi: 10.3390/vaccines9101054 (PMC8539892; doi:10.3390/vaccines9101054)
Supplement: Supplementary file 1 [file vaccines-09-01054-s001.zip › Supplementary Figure S3.pdf]

## Supplementary Figure S3

**A**

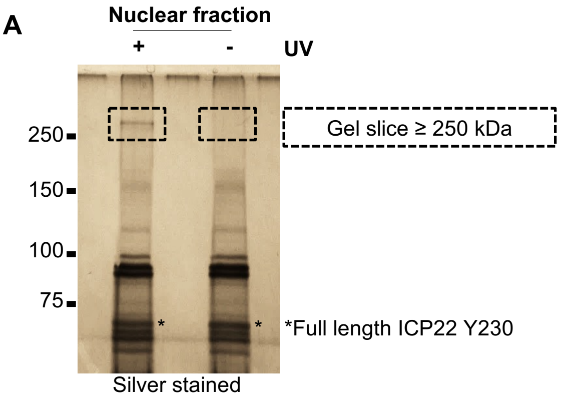


**B**

| Protein band (kDa) | Gene names | Mol. weight [kDa] | REPLICATE 1 | | | | | | REPLICATE 2 | | | | | |
|---|---|---|---|---|---|---|---|---|---|---|---|---|---|---|
| | | | (-UV) | | | (+UV) | | | (-UV) | | | (+UV) | | |
| | | | Peptides | Sequence coverage [%] | Intensity | Peptides | Sequence coverage [%] | Intensity | Peptides | Sequence coverage [%] | Intensity | Peptides | Sequence coverage [%] | Intensity |
| >250 | ICP22 | 46.524 | 6 | 15.7 | 3.44E+08 | 12 | 33.1 | 1.20E+10 | 4 | 11.4 | 2.74E+08 | 8 | 19 | 6.34E+09 |
| | HSP90AB1 | 83.263 | 9 | 15.6 | 5.65E+07 | 21 | 29.6 | 5.87E+08 | 2 | 3.2 | 4.02E+06 | 17 | 24.9 | 4.15E+08 |
| | SUPT16H | 119.91 | 5 | 5.5 | 1.37E+07 | 21 | 22.5 | 2.07E+08 | 1 | 1.1 | 0 | 13 | 14.2 | 9.89E+07 |
| | HSPA8 | 70.897 | 3 | 6.2 | 1.40E+07 | 17 | 28.9 | 2.00E+08 | 0 | 0 | 0 | 14 | 24.5 | 1.53E+08 |
| | PDE4D | 23.839 | 1 | 3.7 | 8.59E+07 | 1 | 3.7 | 1.30E+08 | 0 | 0 | 0 | 1 | 3.7 | 7.42E+08 |
| | HSPA1B;HSPA1A | 70.051 | 4 | 7.6 | 6.27E+06 | 12 | 21.7 | 1.30E+08 | 0 | 0 | 0 | 11 | 18.9 | 1.15E+08 |
| | FAU | 6.6478 | 1 | 16.9 | 3.41E+07 | 2 | 18.6 | 1.10E+08 | 2 | 18.6 | 5.10E+07 | 2 | 18.6 | 7.47E+07 |
| | MKI67 | 319.44 | 0 | 0 | 0 | 1 | 1.2 | 1.01E+08 | 1 | 1.2 | 4.42E+06 | 1 | 1.2 | 4.03E+07 |
| | HSP90AA1 | 84.659 | 3 | 4.9 | 3.60E+06 | 20 | 25 | 9.64E+07 | 2 | 3.1 | 2.90E+06 | 18 | 24.9 | 1.19E+08 |
| | SUMO2 | 8.1111 | 1 | 16.9 | 4.16E+07 | 2 | 31 | 9.20E+07 | 0 | 0 | 0 | 1 | 16.9 | 2.71E+07 |
| | HIST1H4A | 11.367 | 5 | 49.5 | 6.54E+07 | 6 | 50.5 | 9.04E+07 | 3 | 31.1 | 1.68E+07 | 4 | 42.7 | 2.54E+07 |
| | RPS8 | 24.205 | 3 | 14.9 | 2.91E+07 | 5 | 26 | 7.79E+07 | 0 | 0 | 0 | 3 | 14.9 | 1.40E+07 |
| | RPL36AL | 12.469 | 4 | 35.8 | 2.66E+07 | 3 | 28.3 | 5.91E+07 | 2 | 16 | 2.09E+06 | 2 | 16 | 3.71E+06 |
| | SSRP1 | 81.074 | 0 | 0 | 0 | 8 | 13.3 | 4.97E+07 | 0 | 0 | 0 | 4 | 6.8 | 6.93E+06 |
| | RPL18 | 18.091 | 3 | 21.4 | 1.12E+07 | 5 | 36.5 | 4.94E+07 | 1 | 6.3 | 9.60E+05 | 2 | 14.5 | 5.51E+06 |
| | RPL27A | 16.561 | 2 | 14.2 | 1.74E+07 | 3 | 22.3 | 4.44E+07 | 1 | 7.4 | 4.84E+06 | 1 | 7.4 | 5.29E+06 |
| | PARP1 | 113.08 | 0 | 0 | 0 | 11 | 14.1 | 4.35E+07 | 0 | 0 | 0 | 3 | 3.6 | 1.20E+07 |
| | NUMA1 | 236.51 | 6 | 3.4 | 1.09E+07 | 18 | 10.2 | 4.07E+07 | 7 | 4.1 | 1.41E+07 | 10 | 5.8 | 2.19E+07 |
| | HIST1H1C;HIST1H1E;HIST1H | 21.364 | 1 | 5.2 | 1.66E+07 | 2 | 12.7 | 3.75E+07 | 1 | 5.2 | 1.40E+06 | 1 | 5.2 | 4.65E+06 |
| | FASN | 273.42 | 0 | 0 | 0 | 13 | 5.9 | 3.45E+07 | 3 | 2.2 | 9.35E+06 | 18 | 9.1 | 8.12E+07 |
| | PRSS3 | 25.924 | 1 | 5.4 | 2.63E+07 | 1 | 5.4 | 3.25E+07 | 1 | 5.4 | 2.54E+07 | 1 | 5.4 | 3.09E+07 |
| | PRKDC | 469.08 | 1 | 0.3 | 1.07E+06 | 10 | 3.1 | 3.10E+07 | 0 | 0 | 0 | 8 | 2.2 | 1.75E+07 |
| | HSPA4 | 94.33 | 0 | 0 | 0 | 7 | 9.9 | 2.50E+07 | 0 | 0 | 0 | 6 | 8 | 2.60E+07 |
| | HSPA6;HSPA7 | 71.027 | 3 | 5.1 | 4.65E+06 | 8 | 13.8 | 2.45E+07 | 0 | 0 | 0 | 5 | 9.2 | 2.10E+07 |
| | RPL4 | 47.697 | 1 | 2.6 | 3.96E+06 | 4 | 11 | 2.01E+07 | 1 | 2.6 | 1.80E+06 | 1 | 2.6 | 3.28E+06 |
| | THOC2 | 182.77 | 0 | 0 | 0 | 6 | 4.6 | 1.85E+07 | 0 | 0 | 0 | 1 | 0.6 | 1.98E+06 |
| | DDX24 | 96.331 | 0 | 0 | 0 | 6 | 8.7 | 1.77E+07 | 0 | 0 | 0 | 1 | 1.5 | 1.20E+06 |
| | PRPF8 | 273.6 | 0 | 0 | 0 | 6 | 2.9 | 1.43E+07 | 0 | 0 | 0 | 4 | 2.4 | 1.73E+07 |
| | HSPA5 | 72.332 | 0 | 0 | 0 | 2 | 4 | 1.31E+07 | 0 | 0 | 0 | 1 | 2.4 | 9.19E+06 |
| | ST13;ST13P5;ST13P4 | 41.331 | 0 | 0 | 0 | 3 | 10.6 | 1.17E+07 | 0 | 0 | 0 | 1 | 3.8 | 7.98E+06 |
| | CAD | 242.98 | 0 | 0 | 0 | 4 | 2.1 | 8.35E+06 | 2 | 1 | 3.53E+06 | 3 | 2.2 | 8.28E+06 |
| | GTF3C1 | 236.28 | 0 | 0 | 0 | 4 | 1.8 | 7.26E+06 | 0 | 0 | 0 | 4 | 2.5 | 8.38E+06 |
| | NONO | 43.866 | 0 | 0 | 0 | 3 | 9.2 | 6.52E+06 | 0 | 0 | 0 | 1 | 3.1 | 3.05E+06 |
| | HNRNPU | 88.979 | 0 | 0 | 0 | 2 | 4.1 | 6.41E+06 | 0 | 0 | 0 | 1 | 1.9 | 1.81E+06 |
| | TRIM28 | 79.473 | 0 | 0 | 0 | 2 | 6.8 | 6.38E+06 | 0 | 0 | 0 | 1 | 2.7 | 1.71E+06 |
| | C2CD2L | 76.18 | 1 | 1.4 | 5.97E+06 | 1 | 1.4 | 6.23E+06 | 0 | 0 | 0 | 1 | 1.4 | 2.22E+07 |
| | RPL24 | 17.779 | 1 | 5.1 | 2.99E+06 | 1 | 5.1 | 5.59E+06 | 0 | 0 | 0 | 1 | 5.1 | 1.46E+06 |
| | SFPQ | 76.149 | 0 | 0 | 0 | 2 | 4.2 | 5.25E+06 | 0 | 0 | 0 | 1 | 2.4 | 4.81E+06 |
| | RPS6 | 28.68 | 3 | 13.7 | 6.21E+06 | 2 | 8.8 | 4.86E+06 | 0 | 0 | 0 | 1 | 4.8 | 1.71E+06 |
| | RPL13 | 24.261 | 1 | 5.2 | 1.25E+06 | 2 | 11.4 | 4.82E+06 | 0 | 0 | 0 | 1 | 5.2 | 9.24E+05 |
| | TOP1 | 90.725 | 0 | 0 | 0 | 1 | 1.6 | 4.26E+06 | 0 | 0 | 0 | 1 | 1.6 | 1.84E+06 |
| | RRP12 | 132.71 | 0 | 0 | 0 | 2 | 1.6 | 3.29E+06 | 0 | 0 | 0 | 1 | 0.8 | 1.90E+06 |
| | HSPH1 | 92.115 | 0 | 0 | 0 | 2 | 3.3 | 3.25E+06 | 0 | 0 | 0 | 3 | 4.1 | 5.61E+06 |
| | SUMO1 | 11.557 | 0 | 0 | 0 | 2 | 24.8 | 1.68E+06 | 0 | 0 | 0 | 1 | 11.9 | 2.26E+06 |
| | MYBBP1A | 148.85 | 1 | 0.8 | 5.50E+05 | 2 | 2.2 | 1.21E+06 | 0 | 0 | 0 | 2 | 1.4 | 3.28E+06 |
| | EIF4G1 | 154.8 | 0 | 0 | 0 | 2 | 2.9 | 1.07E+06 | 0 | 0 | 0 | 3 | 3.8 | 7.91E+06 |
| | UTP20 | 318.38 | 1 | 0.4 | 8.48E+05 | 1 | 0.4 | 1.05E+06 | 0 | 0 | 0 | 1 | 0.4 | 6.56E+05 |
| | SEC16A | 228.87 | 0 | 0 | 0 | 1 | 0.5 | 6.37E+05 | 0 | 0 | 0 | 1 | 0.5 | 6.94E+05 |

(A) Indicated protein bands (>250kDa) of silver-stained SDS-PAGE gel were excised, in-gel digested and analysed by mass spectrometry. (B) Mass spectrometric analysis of ICP22 (green shade), SPT16 (gene name; SUPT16H) and SSRP1 (blue shades) identified from the silver-stained gel slice of ≥250 kDa of no UV and UV-treated samples. Details of the proteomic data including the untransfected samples can be found in Supplementary Table 4.
