# Supplementary material for: HSV-1 ICP22 Is a Selective Viral Repressor of Cellular RNA Polymerase II-Mediated Transcription Elongation
Source: Vaccines (Basel). 2021 Sep 22;9(10):1054. doi: 10.3390/vaccines9101054 (PMC8539892; doi:10.3390/vaccines9101054)
Supplement: Supplementary file 1 [file vaccines-09-01054-s001.zip › Supplementary Figure S4.pdf]

Supplementary Figure S4

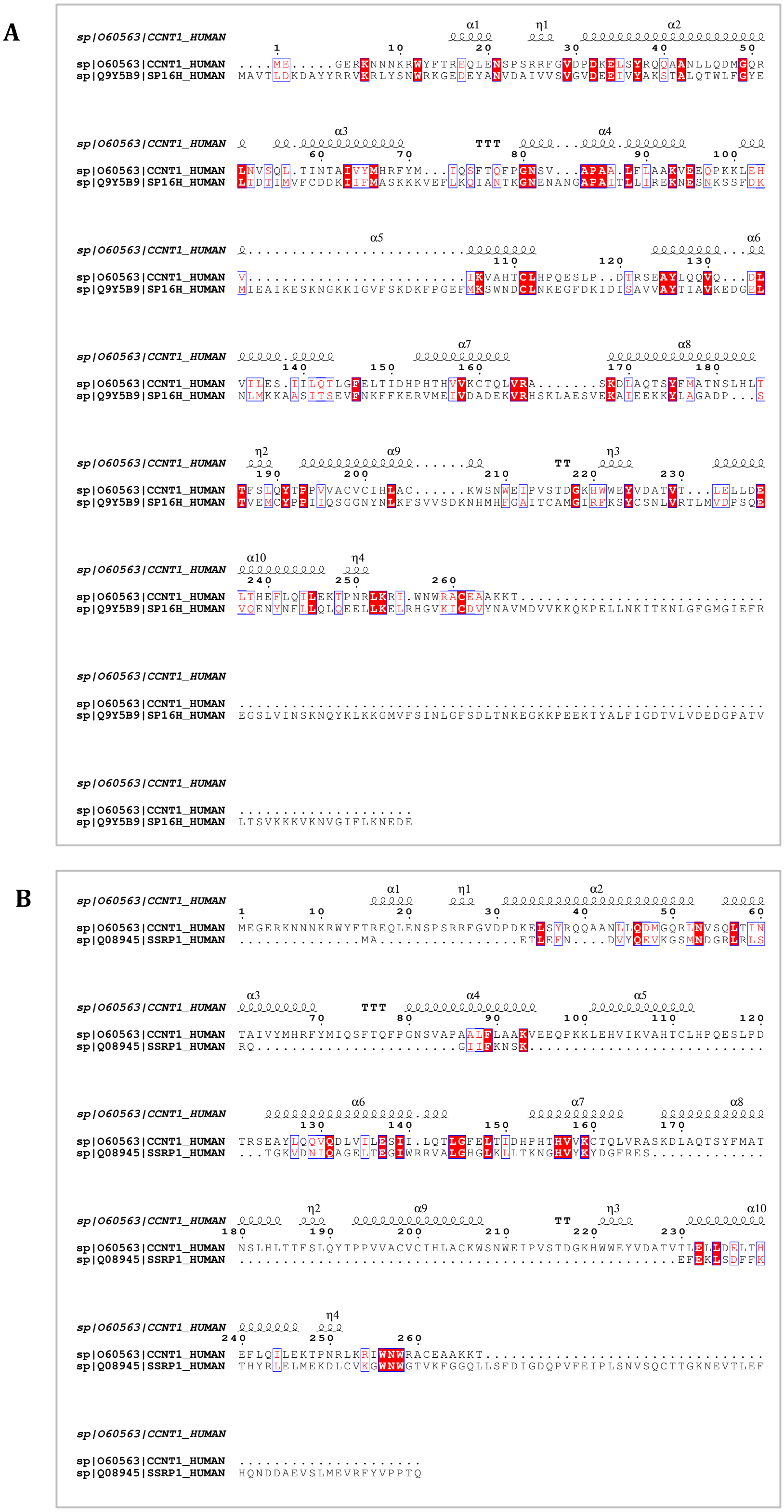

Sequence alignment of Cyclin T1 residues 1-266 (Uniprot ID O60563) and N-terminal region of (A) SPT16 (Uniprot ID Q9Y5B9) or (B) SSRP1 (Uniprot ID Q08945) showing minimal sequence similarity between the two known ICP22-binding complexes.
